# Natural Products in Epilepsy—the Present Situation and Perspectives for the Future

**DOI:** 10.3390/ph3051426

**Published:** 2010-05-12

**Authors:** Dana Ekstein, Steven C. Schachter

**Affiliations:** Department of Neurology, Beth Israel Deaconess Medical Center and Harvard Medical School, 330 Brookline Avenue, BA 504, Boston, MA 02215, USA; E-Mail: sschacht@bidmc.harvard.edu (S.C.S.)

**Keywords:** epilepsy, natural products, complementary and alternative medicine (CAM), botanicals

## Abstract

More efficacious and better tolerated treatments for epilepsy are clearly needed. Complementary and alternative medicine (CAM) has a long history of use in certain parts of the world and has gained increasing interest over the last decades in Western countries. In countries with a Western-based type of medical system, people with epilepsy (PWE) take natural products or engage in other forms of CAM mainly to enhance general health, but also to prevent seizures or to alleviate symptoms of comorbidities or side effects of antiepileptic medications. In other countries, well developed medical systems, such as traditional Chinese Medicine and Ayurveda, are often the basis for treating PWE. Based on anecdotal reports of efficacy in PWE, natural products from these and other traditions are increasingly being studied in animal models of epilepsy, and candidates for further clinical development have been identified. It is likely, therefore, that natural products will be further evaluated for safety, tolerability and efficacy in PWE with drug-resistant seizures.

## 1. Introduction

Approximately one third of people with epilepsy (PWE) have drug-resistant seizures [1,2]. Surgery is highly effective and safe for selected patients with treatment-resistant focal epilepsy [3,4], but is still underused, even in high-income countries [5]. Many PWE may not be candidates for surgery because a single site of origin of their seizures cannot be localized or exists within eloquent regions of the cortex. Other treatment strategies are primarily palliative (vagal nerve stimulation) or still under investigation (closed loop cortical stimulation) [6]. Although the newer antiepileptic drugs (AEDs) may offer a better adverse events profile in comparison to the older generation AEDs, they may still have significant undesired CNS effects such as decreased cognitive abilities and psychiatric complications [7]. Notwithstanding these limitations, the ease of use and ready availability of medications, as well as the prompt reversibility of dose-related side effects, will keep AEDs as the mainstay of epilepsy treatment for the foreseeable future. Therefore, new drug therapies with efficacy against drug-resistant seizures, favorable adverse events profiles, especially in regard to neurological and psychiatric effects, and, if possible, low costs to patients and high worldwide availability are clearly needed.

Complementary and alternative medicine (CAM) is defined by the National Center for Complementary and Alternative Medicine (NCCAM) as a group of diverse medical and health care systems, practices, and products that are not generally considered part of conventional medicine as practiced in the west (e.g. the United States) [8]. Four domains of practices are recognized: mind-body medicine (meditation, prayer, mental healing, art, music, dance), biologically-based practices (use of substances found in nature such as herbs, foods, vitamins, animal compounds), manipulative and body-based practices (chiropractic or osteopathic manipulation, massage), and energy medicine (biofield and bioelectromagnetic therapies). Separately recognized are whole medical systems, such as homeopathy, naturopathy, Ayurveda and traditional Chinese medicine (TCM), each of them characterized by a complex and unique system of diagnostics and therapeutics. For example, the practitioners of TCM use herbal medicine, acupuncture and moxibustion as therapeutic methods. 

The use of different types of CAM by PWE has been comprehensively summarized [9]. Despite the large amount of published information on the use of different CAM interventions for epilepsy, the efficacy of these approaches has not been adequately proved in clinical trials. For example, three Cochrane reviews evaluated the published data on the use of acupuncture [10], yoga [11], and TCM [12] for epilepsy and none found sufficient evidence of efficacy of any of these techniques. Indeed, publications of CAM clinical trials for the treatment of epilepsy that have encouraging results generally use inadequate methodologies. Many do not adequately randomize study subjects or use proper controls, while others are not blinded or do not rigorously monitor the results of the interventions. While the requirements of evidence-based medicine have become familiar to conventional/western practitioners, incorporating these principles into trials of other medical systems is problematic because these systems may involve a holistic, personalized approach to treating patients rather than one that is disease focused and therefore applied in the same way to all patients characterized by a specific disease. 

In this article we focus on one specific CAM treatment for the treatment of epilepsy—non-vitamin, non-mineral natural products, and especially herbal extracts. We first review information about their current use by people with epilepsy around the world and then summarize available data on the efficacy and risks of some of these products. We conclude with possible future directions for natural products as epilepsy therapies.

## 2. Use of Natural Products in Countries with Western/Conventional Medical Systems

In the 11th century, a more unified system of medicine emerged and was thereafter developed into what we today refer to as conventional or Western medicine, which is the principal doctrine underlying the diagnosis and treatment of diseases in North America, Europe, Near East, and Australia. This system emphasizes a disease-focused approach to diagnosis and an evidence-based approach to treatment. While natural products had been widely used as treatments in western medicine for hundreds of years, over the past century, in parallel with the development of the pharmaceutical industry and government regulatory agencies, single compound therapies have predominated in medical practice. This development reflects the evolving philosophy of Western medicine and requirements of regulatory agencies that treatments must have scientifically proven safety and efficacy in the target population. However, over the past few decades, the use of natural products by patients in these countries has significantly increased, driven by their search for “natural”, more effective and less toxic treatments and enabled by improved worldwide communication. These products are typically marketed as dietary supplements and often originate from established medical systems such as TCM or Ayurveda. 

According to the 2007 American National Health Interview Survey of 23,393 adults and 9417 children, 38.3% of adults and 11.8% of children reported using CAM therapies [13]. These therapies were used more frequently by people with higher levels of education, women and the Native American population. The most commonly utilized form of CAM was natural products, which were used by 17.7% of the surveyed adults. Concordantly, the annual out-of-pocket cost in the US for purchase of natural products for 2007 was estimated to be $14.8 billion, which is one third of the out-of-pocket amount spent on prescription drugs [14]. The frequency of use of specific natural products in developed countries appears to parallel the available evidence supporting their use. Despite an overall minor increase from 2002 to 2007 in the estimated frequency of CAM use by American adults (from 36% to 38.3%), there was a marked change in the particular natural products most widely used between these two surveys. The survey performed in 2007 found that the most commonly used products were fish oil/omega-3 fatty acids (37.4%), glucosamine (19.9%), echinacea (19.8%), flaxseed (15.9%) and ginseng (14.1%); whereas according to the 2002 survey, echinacea was used by 40.3%, ginseng by 24.1%, *Gingko biloba* 21.1%, garlic 19.9% and glucosamine 14.9%. These trends generally parallel findings in the western medical literature. For example, a 2000 Cochrane review concluded that echinacea preparations from aerial parts of the plant were effective for the treatment of the common cold [15], while an update published in 2006 expressed much more equivocal conclusions [16]. Similarly, dietary supplementation with omega-3 fatty acids has recently become a mainstream recommendation for many medical conditions and especially cardiac disease [17], following several studies reporting decreased mortality in treated patients [18,19,20]. Not surprisingly, then, fish oil/omega-3 fatty acids was the most-often used natural product in the 2007 survey [13]. 

Eleven population or hospital-based studies have investigated CAM use by adults [21,22,23,24,25,26,27,28] and children [29,30,31] with epilepsy in high-income countries (Table 1). According to these studies, between 24 and 56% of the adult patients and 12 to 32% of children with epilepsy have used CAM therapies at some time. Although only 2 to 44% of these patients reported using these products specifically for control of seizures, the reasons noted by many patients may be relevant to known comorbidities of epilepsy such as depression or to common AED adverse events such as impaired memory. Some of the differences in the frequency of CAM use between studies may pertain to differences in the definition and types of CAM included in each study. However, another possible factor could be inclusion of patients with different ethnicities and cultural backgrounds, as exemplified by studies of patients originating from south Asia in the UK [32] and an ethnically diverse population in Brooklyn, New York [33]. These ethnic and cultural differences could influence the frequency of CAM use as well as the types of CAM used [34]. 

Seven of the above mentioned studies reported the use of specific natural products in their study population, six of them exclusively in PWE (Table 1). We performed a Medline search on the characteristics of all 35 mentioned products, in regard to main uses, adverse events and potential for drug interactions, as well as known or presumed effects on seizures and on AEDs and report our findings in Table 2. Of the six reports, five included more specific numerical data on the use of natural products. We were therefore able to calculate percentage of overall estimates of use in PWE. Although integrating information from studies performed on different populations and over the course of 10 years has clear methodological limitations, this estimate may provide helpful information for physicians who treat PWE. 

The three most frequently taken products were ginseng (reported by 17%), Gingko biloba (16%) and St. John’s wort (13%). This is interesting because these extracts are generally used for amelioration of symptoms of anxiety, depression and memory deficits, which are commonly encountered comorbidities of epilepsy [35]. While all three herbs have been reported to have beneficial effects on seizures, it is concerning that each has been reported to aggravate seizures as well. Interestingly, in the case of Gingko biloba, there is evidence to suggest that part of the plant may be epileptogenic (the seeds) while other parts (the leaves and the stem) may protect against seizure activity [36]. In contrast, the effect of St. John’s wort on seizures may depend on the extraction method [37]. Gingko biloba and St. John’s wort [38] may also have clinically relevant interactions with hepatically-metabolized AEDs.

The next most frequently used products in this population of PWE were echinacea (11%), garlic (10%), cranberry (9%) and soy (8%). These are commonly used in the general population as so-called immune enhancers (echinacea, garlic) and for prevention and ameliorations of symptoms of urinary tract infections (cranberry) and menopause (soy). The higher percentages of use in PWE compared to the general US population [13] could potentially be explained by a lower self perceived level of general health among PWE, as previously suggested [39]. These products have not been reported to have either beneficial or detrimental effects on seizures. However, their presumed effects on the P450 system could potentially lead to interactions with AEDs metabolized by the liver.

Products used by 2 to 7% of PWE in the studies cited earlier include substances that may have specific benefits for this population. Sleep induction and anticonvulsant effects have been reported for melatonin [40], kava kava [41] and valerian [42], though melatonin [43] and kava kava [41] have also been associated with aggravation of epilepsy. Evening primrose is used for alleviation of menopausal symptoms, and some of its compounds have mechanisms of actions consistent with anticonvulsant properties [44]. 

**Table 1:** Publications reporting on use of CAM in countries with western style medical system.

Publication	Studied population	% using CAM	Associations	Doctor’s knowledge	Most used CAM (in descending order, where known)	Reason for use	Outcome measures	Most used non-vitamin, non-mineral natural products (in descending order, where known)
Gidal *et al.* ,1999 [21]	465 adults with epilepsy from 9 regions in the US	31%, within previous year	Associated with high-school education or less. No influence of age, gender, seizure type.	33%	*Ginkgo biloba*, vitamins (55%), relaxation (45%), ginseng, St. John’s wort	13% epilepsy (relaxation, vitamins, herbals, homeopathy); 28% general health/cold prevention; 11% mood difficulties; 5% cognition; 4% fatigue.	NA	Gingko biloba (81% of users; 63% used it for cognition), ginseng (44% of users), St. John’s wort (used by 7% of pts.; 53% for mood and 24% for fatigue)
Peebles *et al.*, 2000 [22]	92 adults with epilepsy in Ohio (US)	24%	No significant association with education level, sex, ethnicity, age	31%	Massage (50%), herbs/supplements (41%), music therapy, meditation, art therapy, aromatherapy, acupuncture	2% epilepsy (massage, acupuncture and meditation); pain; muscle tension; stress; low energy; cold; depression.	NA	4 Ginseng, 3 St. John’s wort, 3 melatonin, 3 Ginkgo biloba,1 garlic, 1 black cohosh
Waaler *et al.*, 2000 [29]	198 children with active epilepsy in Norway	11.60%	Additional neurological deficits	NA	Homeopathy	NA	NA	NA
Gross-Tsur *et al.*, 2003 [30]	115 children with epilepsy in Israel (compared with children with ADHD and control)	32%	In general - higher education, prior use for current use; for epilepsy and ADHD - longer disease duration, less satisfaction with conventional therapy	NA	Dietary interventions most and also homeopathy, biofeedback, acupuncture, Reike, reflexology, Shiatsu, chiropractice (in all groups)	NA	NA	NA
Sirven *et al.*, 2003 [23]	425 adults with epilepsy in Arizona (US)	44% for epilepsy, 42% for other conditions	No association with education level	93% would tell	Prayer, stress management, botanicals, chiropractic (specifically for epilepsy)	44% epilepsy, 42% other conditions	Stress management, yoga and botanicals subjectively most beneficial. 43% using botanicals for epilepsy had increased seizure frequency; 3 had major side effects (intracranial hemorrhage with ephedra).	General/epilepsy use: 76/13 Garlic, 157/12 gingko, 64/10 soy, 40/11 melatonin, 22/10 kava, 159/8 ginseng, 147/9 St. John’s wort, 89/9 Echinacea, 68/3 cranberry, 40/5 goldenseal, 24/7 grapeseed, 21/4 black cohosh, 33/4 valerian, 14/3 saw palmetto, 7/7 evening primrose, 12/2 licorice, 7/4 hops,3/2 black haw
Plunkett *et al.*, 2004 [24]	187 adults with epilepsy in San Francisco area (US)	56%	No association with seizure frequency or with having adverse events from AED.	68%	Vitamins or minerals supplements	3% epilepsy or AED adverse events; general health; supplementing diet; physician’s recommendation.	NA (19% used products wit cyp450 activity and 14% - potentially epileptogenic agents.)	Garlic, echinacea, St. John’s wort, ephedra, ginseng, gingko, evening primrose
Yuncker *et al.*, 2004 [31]	350 children with neurological conditions (60% had epilepsy) in Pennsylvania (US)	28% of children with epilepsy (37% of all conditions)	Diagnosed for less than one year	69%	NA	NA	87% overall felt CAM was effective and similar to conventional therapy. 40% knew possible side effects.	NA
Easterford *et al.*, 2005 [25]	377 adults with epilepsy in Manchester, UK	34.60%	Higher education	37%	NA	11.1% epilepsy	No significant effect on seizure frequency. CAM was cheap.	NA
Liow *et al.* , 2007 [26]	228 adults with epilepsy in mid west US	39%	No association with education level	49%	Prayer/spirituality, megavitamins, chiropractic, stress management	57 (25%) epilepsy: 33 prayer/spirituality; 14 megavitamins; 11 chiropractic; 11 stress management	Subjective benefit of 74% of 57. Only few side effects. Increased szs in diet pills, chiropractic, ketogenic diet, atkin’s megavitamins.	10 Cranberry, 8 black cohosh, 7 Echinacea, 6 melatonin, 4 garlic, 4 grape seeds, 4 soy, 4 St. John’s wort, 4 valerian, 3 evening primrose, 2 Ginkgo biloba, 2 ginseng, 1 black haw
Murphy *et al.*, 2008 [27]	671 adults with neurological conditions in Ireland (189 with epilepsy)	47.6% of patients with epilepsy (63.3% of all conditions)	NA	25% for all conditions	massage, acupuncture, vitamins, reflexology, yoga, evening primrose/starflower oil, chiropractic, homeopathy (for all conditions)	NA	Most had subjective benefit. Annual cost was 1170.32 euro.	97 evening primrose/starflower oil, 24 marijuana, 16 St. John’s wort, 16 Gingko biloba, Udo’s oil, fish oil, black cohosh, echinacea, bonemeal, coenzyme Q-10
Kaiboriboon *et al.*, 2009 (only use of herbs and dietary supplements was studied) [28]	187 adults with epilepsy at UCSF medical center (US)	56% (current use)	Partial epilepsy and Caucasian race. No association with gender, age, level of education, income, duration of epilepsy or seizure frequency.	71%	Multivitamins and minerals, folic acid, ginseng, *Ginkgo biloba*, glucosamine and chondroitin, St. John’s wort, black cohosh, Echinacea, evening primrose, ephedra, caffeine, melatonin, milk thistle, omega 3, kava, skullcap, valerian, grapefruit juice, glutamine, clover/nettles, parsley leaf, DHEA, Coenzyme Q10, ginger, fish oil, garlic, grape seed, L-lysine	6 epilepsy (kava, skullcap, valerian, folic acid, vit. B6, vit. E, multivitamins, minerals); 35 general health; 13 physician’s recommendation; 13 improve bone density; 10 increase energy; 10 boost immune system; 7 improve memory.	9 patients reported adverse events that they attributed to these products. None reported aggravation of seizures. 88% of patients spent less than $50 a month and only 5% spent more than $100.	4 Ginseng, 4 Ginkgo biloba, 4 glucosamine and chondroitin, 3 St. John’s wort, 2 black cohosh, 2 Echinacea, 2 evening primrose, 2 ephedra, 2 caffeine, 2 melatonin, 2 milk thistle, 2 omega 3, 1 kava, 1 skullcap, 1 valerian, 1 grapefruit juice, 1 glutamine, 1 clover/nettles, 1 parsley leaf, 1 DHEA, 1 Coenzyme Q10, 1 ginger, 1 fish oil, 1 garlic, 1 grape seed, 1 L-lysine

CAM—complementary and alternative medicine; NA—not available; AED—anti-epileptic drug.

**Table 2:** Characteristics of natural products used by patients with epilepsy in countries with western based type of medical system.

Product	Main current medical uses	Main adverse events & interactions	Overall estimated extent of use in PWE*	Possible effects in epilepsy	Potential risks for PWE
Black cohosh (*Cimicifuga racemosa*) [22,23,26,27,28^$^]	Ameliorates menopausal symptoms	Possible hepato-toxicity	4%	NR	NR
Black haw (*Viburnum prunifolium*) [23,26]	Spasmolytic, sedative, anti asthmatic	NR	<1%	NR	NR
Bonemeal [27]	Calcium supplementation (not used recently)	Possible prion infection		NR	NR
					Seizures secondary to lead poisoning
Caffeine [28^$^]	Stimulant, antinociceptive	Sympathomimetic and GI symptoms	<1%	NR	Associated with seizures. May interact with CBZ.
Chondroitin [28^$^]	Anti arthritis & arthralgia	GI symptoms, hypersensitivity, possible anticoagulation (increased INR with Warfarin)	<1%	NR	NR
(Red) clover (*Trifolium pretense*) [28^$^]	Ameliorates menopausal symptoms	Possibly inhibits aromatase and extrahepatic CYP1A1, 1B1. May increase INR when taken with Warfarin	<1%	NR	NR
Coenzyme Q10 [27,28^$^]	Antioxidant, ameliorates CHF and neurodegenerative disorders	May increase risk of bleeding in Warfarin users	<1%	May improve seizures in neurological mitochondrial diseases	NR
Cranberry [23,26]	Prevents UTI, cardioprotector, anti-cancer, antioxidant	GI intolerance, weight gain, possibly thrombocytopenia and increase in INR in combination with Warfarin	9%	NR	NR
					Inhibited P450 system *in vitro*, but not *in vivo*.
DHEA [28^$^]	Hormonal replacement therapy in elderly women and men	No adverse events. Induces CYP2B6	<1%	NR	Possibly associated with seizures
				No effect in animals	
Echinacea [23,24,26,27,28^$^]	Enhances immune system functions	Hypersensitivity, GI disturbance, hepatotoxicity with prolonged use; mild effect on CYP3A4 and CYP1A2	11%	NR	NR
Ephedra [24,28^$^]	Induces weight loss, stimulant	HTN, tachycardia, stroke	<1%	NR	Associated with seizures
Evening primrose (*Oenothera biennis*) [23,24,26,27,28^$^]	Ameliorates menopausal symptoms	NR	2%	Anti-seizure effects (animals)	Possibly associated with seizures
Garlic [22,23,24,26,28^$^]	Antioxidant, cardiovascular, cancer prevention, immune stimulant	GI disturbance, dizziness. Possible increase in INR, HTN, arrhythmia. Possible influence on CYP3A4 and inhibitor of CYP2C9/19	10%	NR	NR
Ginger [28^$^]	Prevents cardiovascular disease and cancer, ameliorates GI symptoms, antioxidant, anti-inflammatory	May increase risk of bleeding in warfarin users. Possible inductor of CYP1A2 and 3A4	<1%	May have antiepileptic effects	NR
Ginseng [21,22,23,24,26,28^$^]	Anxiety, depression, concentration problems, DM, stimulant, menopausal symptoms, sexual dysfunction in men	Possible hepatotoxicity & increased INR, lethargy	17%	May have anticonvulsive properties	May exacerbate seizures
Ginkgo biloba [21,22,23,24,26,27,28^$^]	Cognitive enhancer, prevents cardiovascular disease, antioxidant	GI discomfort, anti-coagulant. May inhibit CYP2B6 and induce CYP3A & 1A2, 2C19.	16%	Leaves and stems may be anticonvulsive	Seeds may be toxic and pro-convulsive. May increase metabolism of AED (PHT, VPA, PB)
Glucosamine [28^$^]	Anti-arthritis & arthralgia	May increase INR in warfarin users.	<1%	NR	NR
Glutamine [28^$^]	Enhances healing after injury, especially GI, promotes muscle building	NR	<1%	NR	NR
Goldenseal (*Hydrastis Canadensis*) [23]	Anti-inflammatory, anti-microbial	Possible hypernatremia, HTN, edema. Strong inhibitor of CYP2D6 & CYP3A4/5, CYP2E1	5%	NR	NR
Grapefruit juice [28^$^]	Prevents cardiovascular disease and cancer, antioxidant	Inhibits enteric CYP3A4, possibly increases risk of breast cancer	<1%	NR	Increases bioavailability of CBZ, BDZ
Grape seeds [23,26,28^$^]	Prevents cardiovascular disease and cancer, antioxidant	Hypersensitivity, HA, dizziness, nausea. May inhibit CYP2E1	4%	NR	NR
Hops (*Humulus lupulus*) [23]	Ameliorates anxiety, insomnia and menopausal symptoms, anti-inflammatory, antioxidant	Sedation. Possibly inhibits CYP2C9, 1A1/2, 1B1	<1%	NR	Possibly exacerbates seizure
Kava kava (*Piper methysticum*) [23,28^$^]	Ameliorates depression, anxiety and insomnia, induces local anesthesia	Possibly psychosis, choreoathetosis, hepatotoxicity, dermopathy, lymphopenia, GI disturbances, potential for addiction. Inhibitor of CYP2E1, 1A2, induction of 1A1.	4%	May benefit	Possibly exacerbate seizures
L-lysine [28^$^]	Precursor of carnitine, promotes protein building, anti-cancer, anti-viral	NR	<1%	NR	NR
Licorice (liquorice or *Glycyrrhiza glabra*) [23]	Expectorant, anti-viral, anti-spasmodic, ameliorates gastric & duodenal ulcers, and menopausal symptoms	In overdose - hypokalemia and increase in BP, decrease testosterone in men. May increase INR when taken with warfarin. Possibly inhibits CYP3A4, 2B6, 2C9.	2%	NR	NR
Marijuana (*Cannabis sativa*) [27]	Antinociceptive, ameliorates glaucoma, anti-nausea, muscle relaxant, promotes bone health	Tachycardia, dry mouth, red eyes, euphoria, anxiety, psychosis, memory deficit. Possibly induces CYP2E1		May benefit	May exacerbate seizures
Melatonin [22,23,26,28^$^]	Ameliorates insomnia, circadian rhythm abnormalities, depression, and anxiety	HA, nausea, somnolence	7%	May benefit	Associated with increased seizure frequency
Milk thistle [28^$^]	Ameliorates hepatotoxicity and DM, anticancer	Possible downregulation of CYP3A4, 2C9	<1%	NR	NR
Nettles (*Urtica dioica*) [28^$^]	Antinociceptive, anti-arthritis, antioxidant, diuretic, ameliorates BPH	Uterine stimulant	<1%	NR	NR
Omega 3/fish oil [27,28^$^]	Cardioprotective, ameliorates depression, dementia, ADHD	Anti-coagulant	<1%	May be beneficial (SUDEP)	NR
Parsley leaf [28^$^]	Diuretic, ameliorates constipation, promotes bone health	May increase INR when taken with warfarin. Possibly downregulates p450. Possibly stimulates uterine contractions.	<1%	NR	NR
Saw palmetto (Serenoa repens) [23]	Ameliorates UTI and BPH	GI disturbance, HA, decreased libido. Inhibition of CYP3A4, 2D6, 2C9.	2%	NR	NR
Skullcap (Scutellaria) [28^$^]	Sedative, anti-epileptic, hypnotic, anxyolytic, anti-cancer	NR	<1%	May benefit	NR
Soy [23,26]	Source of Omega 3 & 6 fatty acids. Ameliorates menopausal symptoms, prevents prostate cancer, and promotes bone health	May increase risk of developing breast cancer. Possibly inhibit CYP2A6, 1A1, 1B1, 3A4.	8%	NR	NR
St. John’s wort (Hypericum perforatum) [21,22,23,24,26,27,28^$^]	Ameliorates depression, pain, ADHD	Possibly hepatotoxicity, visual disturbances, paresthesia, myalgia, GI disturbances, sedation, photosensitivity. Inducer of CYP3A4, 2E1, 2C19, P-glycoprotein. Possible inhibition of CYP2C9/19, 2D6, 3A4, 1A2. Avoid MAO inhibitors and SSRIs.	13%	Possible benefit	Possibly associated with seizures
Valerian [23,26,28^$^]	Ameliorates anxiety, insomnia, and epilepsy, nociceptive	GI disturbance, drowsiness, HA. Avoid barbiturates. Possibly inhibits CYP2D6, 3A4	5%	May benefit	NR

* The overall extent of use was calculated by combining data from the publications that provided numerical data for the use of specific products. For Ginseng, *Gingko biloba* and St. John’s wort, the information was taken from the publications by Gidal, Peebles, Sirven, Liow, and Kaiboriboon (on a total of 1407 PWE) and for all other products from the publications by Peebles, Sirven, Liow, and Kaiboriboon (on a total of 942 PWE) (see last column of Table 1 for enumeration of use of natural products). ^$^This reference reports on neurological conditions in general, including epilepsy. PWE—people with epilepsy; NR—not reported; GI—gastro-intestinal; CHF—congestive heart failure; UTI—urinary tract infection; HTN—hypertension; BHP—benign hypertrophy of prostate; SUDEP—sudden unexplained death in epilepsy patients; HA—headache; AED—anti-epileptic drugs; PHT—phenytoin; VPA—valproic acid; PB—phenobarbital; CBZ—carbamazepine; BDZ—benzodiazepines.

Other infrequently used products mentioned in the studies that have anecdotally been reported to have beneficial effects on seizures, epilepsy comorbidities or complications of epilepsy are skullcap (anticonvulsant, sedative [45]), grapefruit juice (sedative [46]), hops (sedative [47]), and omega-3 fatty acids (treatment of epilepsy and prevention of SUDEP [48]). However, others have been linked to pro-convulsive effects, such as ephedra and caffeine [49]. In addition, grapefruit juice is a potent inhibitor of the enteric P450 system, therefore increasing the bioavailability of many drugs, including certain AEDs [46]. 

The efficacy of natural products for control of seizures cannot be accurately assessed from the available studies because of their significant methodological shortcomings. For example, only one study assessed efficacy using a generally accepted method, *i.e.* the medical notes of treating physicians [25]. However, patients typically report overall subjective benefit in these studies; whether this is consistent with the general population’s view of CAM as “natural” and therefore beneficial requires further study. 

Because of the risk of pharmacokinetic interactions with AEDs, it is concerning that many patients do not inform their physicians about their use of natural products [21,22,25,26,27]. Two publications noted that treatment with natural products was generally widely affordable [25,28], whereas a third one reported on a relatively high annual cost of 1170 euro [27].

## 3. Use of Natural Products in Countries Where Traditional Medicine Is Widely Practiced

The CAM therapies found in regions with widely practiced forms of traditional medicine are generally much more diverse than in countries with a strong western-based type of medical system. This diversity may arise from the relatively lower financial resources of patients in addition to the availability of and access to traditional medicine systems. For example, in sub Saharan Africa, limited financial resources restrict access to western-style medicine, and most persons live very traditionally, with mystical beliefs influencing their day-to-day lives and affinity for traditional healers. There are notable exceptions, such as high-income countries like South Korea and Japan with well-developed systems of traditional medicine practiced alongside western-style medicine. 

Ayurveda and TCM are among the best known systems of traditional medicine. Ayurveda originated thousands of years ago and continues to be widely practiced in South Asia, especially in India. TCM is widely practiced and carefully regulated by the Chinese government. The Kampo system in Japan, as well as Korean Oriental Medicine (KOM), are derived from TCM and remain broadly similar. 

**Ayurveda**—This is the oldest known system of medicine, developed by the ancient Hindus, and based on balancing the three elements (*vata, pitta, kapha*) in the human body. Epilepsy (*Apasmara*) is considered a mental disease and classified into four types, three with predominant involvement of each of the three elements and a fourth characterized by combined involvement of all three. Although some characteristics of seizures as defined in western medicine also appear in Ayurvedic writings, there are differences in how seizures are recognized between these two systems. Treatment usually starts with drastic cleansing of the body through emesis, enemas and purgatives, followed by different Ayurvedic drug formulations. Additional Ayurvedic drugs are then recommended for use and are administered by various ways: orally, by nasal or ocular application, anointing the body or by fumigation [50].

Ayurvedic practitioners prescribe PWE mixtures of natural products, containing herbal extracts, as well as animal ghee, honey and milk. The most widely used herbal extracts are prepared from *Acacia arabica, Acorus calamus, Bacoppa monnieri, Clitorea turuatea, Celastrus panniculata, Convulvulus pluricaulis, Emblica officinalis, Mukta pishti, Whithania somnifera, and Vaca brahmi yoga* [51].

**TCM**—This medical system concerns the study of human physiology and pathology, and the prevention, diagnosis and treatment of human diseases, and dates back more than 2500 years [52]. The theory of TCM for PWE is difficult to understand from a western perspective because of the TCM approach to diagnosis and treatment, and the related principles of holism and differentiation. Four subtypes of epilepsy are recognized, but, as is the case with the classification of seizures in Ayurveda, they do not match directly to the ILAE classification of seizures and epilepsies. Treatments typically involve mixtures of different herbal extracts (each containing many active compounds), some to directly treat the seizure disorder and others to maintain the general wellbeing of the host. The treatment may be applied in three phases. First, seizures are treated by herbs and acupuncture; then, herbs, acupuncture and moxibustion are used for tonic strengthening of organs; and finally, daily life guidelines are recommended to prevent relapse [52]. 

The most frequently used herbal medicines in the published clinical epilepsy literature from the Far East are *Pinella ternate, Arisaemi japonicum, Acorus calamus, Gastrodia elata, Buthus martensii, Poria cocos, Bombyx bartryticatus, Citrus reticulate, Uncaria rhynchophylla, Glycyrrhiza glaba, Salivae miltiorrhizae, Scolopendra subspinipes, Bupleurum falcatum, Succinum, Paeonia albiflora, Panax ginseng, Perichaeta communissma and Curcuma longa* [51]. However, as mentioned earlier, there is insufficient evidence at the present time to recommend the use of TCM for treatment of epilepsy [12]. Nevertheless, extracts used for seizure control in TCM, as well as single compounds derived from these extracts, are being tested alone and in combinations for anticonvulsant effects in animal models of epilepsy. 

**Sub-Saharan African practices**—In this region of the world, there is no widely practiced traditional medical system, such as TCM. Individual local healers acquire their skills from their teachers of the previous generation and different healers may have different approaches to the diagnosis and healing processes as well as a different array of therapeutic products. Although heredity is acknowledged as an etiologic factor, epilepsy is frequent thought to be contagious or provoked by witchcraft, and burns are considered a sign of intractable disease [53,54]. Some healers only recognize epileptic seizures as those that would be classified in the west as generalized tonic-clonic seizures [53], whereas others may use broader definitions [54]. Treatments are based on spiritual measures and natural products derived from herbs and animals that naturally exhibit behavior resembling convulsions or loss of consciousness.

Tens of botanicals have been reported to be used in the treatment of epilepsy in different parts of sub Saharan Africa [55,56,57], by oral delivery, inhalation and topical application. However, there is inadequate study of these treatments to understand their relative frequency of use, efficacy and adverse events.

## 4. Perspectives for the Future

Western-style clinicians should educate their patients about the potential problems associated with natural products and should encourage their patients to inform them when using these products, so that potential adverse effects on seizures and plasma concentrations of AEDs can be monitored. In addition, symptoms of comorbid conditions may come to light and perhaps be more effectively treated. Counseling patients requires that clinicians become familiar with the natural products commonly used by their patients. Patients should understand the current differences in manufacturing of dietary supplements and pharmaceuticals. In the US, herbal medicines have been regulated by the 1994 Dietary Supplement and Health Education Act, which did not require manufacturers to use good manufacturing practice (GMP) standards, which is the case for pharmaceuticals. However, in 2007 the FDA issued “current good manufacturing practice in manufacturing, packaging, labeling, or holding operations for dietary supplements” [58]. This new regulation will prevent significant variation from lot to lot, bottle to bottle, or pill to pill for any dietary supplement [59]. 

In developing countries, natural products may be more accessible, culturally acceptable and affordable then “western” medicines. Therefore, there is a need to scientifically study the safety and efficacy of natural products that are available and recommended by local healers in these regions. The principles of evidence-based medicine apply to these products just as much as they do for single compounds.　 

Indeed, the publication of FDA guidelines for the approval of herbal products for specific indications [60] has sparked an interest in developing strategies for systematically evaluating the efficacy and safety of these products, and increases the potential for industry to invest in their clinical development. In the case of epilepsy therapies, evaluation of candidate products in animal models of epilepsy should precede clinical studies, as is done with pharmaceuticals. Notably, there was a more than 4-fold increase in the number of papers found on PubMed reporting pre-clinical studies of herbal products or their derivatives from 9 in 1998–1999 to 37 in 2008–2009. Some studies report *in vivo* effects and others on mechanisms of action. 

A recent article grouped the new investigational drugs for epilepsy into several categories [61]: i. modifications of existing AEDs to enhance their efficacy and safety; ii. New molecular targets and classes of drugs; derived from knowledge of the mechanisms underlying epilepsy and epileptogenesis; iii. Miscellaneous substances first found to have antiepileptic activity and subsequently being evaluated for their mechanisms of action. The investigation of natural products for use in epilepsy has proceeded using the second and third strategies. One such approach is to test natural products for actions relevant to known mechanisms of actions of AEDs. This approach has the advantage of being able to screen a large number of products at once. At the request of healers, Awad *et al.* tested 34 plants used by the Q’eqchi’ Maya to treat epilepsy and anxiety for their ability to inhibit gamma aminobutyric acid transaminase (GABA-T) and to bind to the benzodiazepine (BDZ) site of the GABA A receptor [62]. At the highest tested concentrations, they found greater than 50% inhibition of GABA-T by 10 plants and greater than 50% binding to the GABA A receptor by 23 plants. Interestingly, a positive correlation was found between the degree of GABA-T inhibition and frequency of use for epilepsy and between the degree of affinity to the receptor and frequency of use for anxiety, suggesting a plausible scientific basis to their use. However, the correlation coefficients were low, implying that other mechanisms may also be important. The limitation of the approach used in this study is that by design it focuses on a known mechanism of action and therefore is not likely to identify natural products with unique mechanisms of action. 

Another interesting approach is to screen plants for potential activity in models of epileptogenesis [63]. Sucher screened natural products traditionally used in TCM for stroke, and assessed activity at multiple molecular targets in the glutamate-receptor triggered signaling pathway leading to neuronal injury and death. He found that many of the herbal drugs contained different compounds that act with low affinity at multiple molecular targets in the pathway. Since these processes occur in parallel and may be hypothesized to predispose to the development of chronic epilepsy after different types of brain injury, he proposed that their combined synergistic activity may give rise to neuroprotective and, possibly, antiepileptogenic effects, with a good safety profile. 

A third approach, used by the authors of this article, is to: (1) identify herbal therapies and compounds isolated from them that have promising activity in animal epilepsy models and/or relevant *in vitro* assays; (2) conduct the pre-clinical studies necessary to proceed with early stage clinical studies; and (3) plan and initiate these clinical studies. Extracts and mixtures of extracts of herbal therapies, as well as pure compounds isolated from them, are first identified based on clinical recommendations of herbal experts around the world, review of original text references, and published results of laboratory or clinical studies. To date, 30 products have been studied in animal models of epilepsy, pain and neuroprotection through the NINDS Anticonvulsant Screening Project (ASP) and in *in vitro* assays of neuronal receptor or ion channel function. Twenty one of these products (70%) have proven to be active. One compound, huperzine A, has shown potent anticonvulsant and antinociceptive activity in rodent models [64,65] and is the subject of the first clinical trial from our program. 

## 5. Summary

Natural products are widely used by PWE all over the world, but there is currently little evidence of safety and efficacy to scientifically justify their use. Nonetheless, natural products with a long history of medicinal use in epilepsy or relevant mechanisms of action should be further tested using systematic pre-clinical methods and rigorously studied in PWE where appropriate as potential new treatments for epilepsy.
